# Identification of Coinfections by Viral and Bacterial Pathogens in COVID-19 Hospitalized Patients in Peru: Molecular Diagnosis and Clinical Characteristics

**DOI:** 10.3390/antibiotics10111358

**Published:** 2021-11-07

**Authors:** Giancarlo Pérez-Lazo, Wilmer Silva-Caso, Juana del Valle-Mendoza, Adriana Morales-Moreno, José Ballena-López, Fernando Soto-Febres, Johanna Martins-Luna, Hugo Carrillo-Ng, Luís J. del Valle, Sungmin Kym, Miguel Angel Aguilar-Luis, Issac Peña-Tuesta, Carmen Tinco-Valdez, Luis Ricardo Illescas

**Affiliations:** 1 Division of Infectious Diseases, Guillermo Almenara Irigoyen National Hospital-EsSalud, Lima 15033, Peru; adri.mmm93@hotmail.com (A.M.-M.); jose.ballena@upch.pe (J.B.-L.); fernando.soto.f@upch.pe (F.S.-F.); Irillescas@gmail.com (L.R.I.); 2 Centre of Research and Innovation, Faculty of Health Sciences, School of Medicine, Universidad Peruana de Ciencias Aplicadas, Lima 15023, Peru; hugo.carrillo.n@upch.pe (H.C.-N.); miguel.aguilar@upc.pe (M.A.A.-L.); isaacp1503@gmail.com (I.P.-T.); carmenrosatv@gmail.com (C.T.-V.); 3 Laboratorio de Biologia Molecular, Instituto de Investigación Nutricional, Lima 15024, Peru; jo_marlu@hotmail.com; 4 Facultad de Ciencias de la Salud, Universidad Tecnológica del Perú, Lima 15046, Peru; 5 Barcelona Research Center for Multiscale Science and Engineering, Departament d’Enginyeria Química, EEBE, Universitat Politècnica de Catalunya (UPC), 08034 Barcelona, Spain; luis.javier.del.valle@upc.edu; 6 Korea International Cooperation for Infectious Diseases, Chungnam National University College of Medicine, Daejeon 305764, Korea; smkimkor@cnu.ac.kr

**Keywords:** coinfections, COVID-19, antibiotics

## Abstract

The impact of respiratory coinfections in COVID-19 is still not well understood despite the growing evidence that consider coinfections greater than expected. A total of 295 patients older than 18 years of age, hospitalized with a confirmed diagnosis of moderate/severe pneumonia due to SARS-CoV-2 infection (according to definitions established by the Ministry of Health of Peru) were enrolled during the study period. A coinfection with one or more respiratory pathogens was detected in 154 (52.2%) patients at hospital admission. The most common coinfections were *Mycoplasma pneumoniae* (28.1%), *Chlamydia pneumoniae* (8.8%) and with both bacteria (11.5%); followed by Adenovirus (1.7%), *Mycoplasma pneumoniae*/Adenovirus (0.7%), *Chlamydia pneumoniae*/Adenovirus (0.7%), RSV-B/*Chlamydia pneumoniae* (0.3%) and *Mycoplasma pneumoniae*/*Chlamydia pneumoniae*/Adenovirus (0.3%). Expectoration was less frequent in coinfected individuals compared to non-coinfected (5.8% vs. 12.8%). Sepsis was more frequent among coinfected patients than non-coinfected individuals (33.1% vs. 20.6%) and 41% of the patients who received macrolides empirically were PCR-positive for *Mycoplasma pneumoniae* and *Chlamydia pneumoniae*.

## 1. Introduction

Coronavirus disease 2019 (COVID-19), caused by the SARS-CoV-2 virus, was declared a pandemic on 11 March 2020 [1]. COVID-19 represents a major public health threat to Latin America, given that it is considered the most inequitable region in the world according to international indexes [2]. Thus, the pandemic has exposed the income inequalities and lack of access to appropriate health care services in Latin America countries [1]. For instance, the spread of COVID-19 in Peru overwhelmed the unprepared, precarious and fragmented health system [3].

The still unknown impact of coinfection rates between SARS-CoV-2 and other respiratory pathogens added to the rapid global expansion of the virus and its variants requires establishing an efficient and sustainable diagnostic strategy over time [4]. Coinfections rates may be higher than expected, which may pose a great challenge for clinicians in the diagnosis and management of patients [5,6]. Several studies have reported a wide variance of coinfection rates in SARS-CoV-2 patients, ranging from 3% to more than 20% [5].

The most frequent pathogens identified among coinfections are group A *Streptococcus* [7], *Mycoplasma pneumoniae* [8], influenza A [9], parainfluenza [10], rhinovirus, enterovirus, respiratory syncytial virus (RSV) and other coronaviruses [5,11]. Current evidence suggests that coinfections with other respiratory viruses may complicate the disease course, leading to increased disease severity and mortality. Therefore, studies that identify the pathogens that coinfected COVID-19 patients and the evaluation of their impact on the clinical outcome are crucial. This data may guide clinicians to establish a directed antimicrobial therapy, decrease the irrational use of antibiotics and improve the clinical outcome [5]. 

This study sought to identify the respiratory pathogens causing coinfections in patients with moderate/severe SARS-CoV-2 pneumonia from a hospital in Peru and determine the clinical characteristics and clinical outcome of coinfected and non-coinfected patients.

## 2. Results

**Figure 1:**
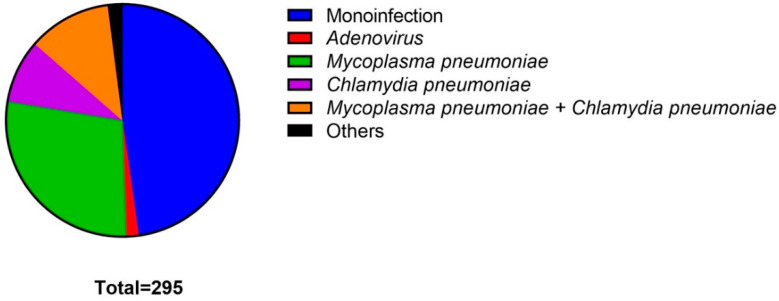
Coinfections reported, showing that 141 (47.8%) patients had SARS-CoV-2 as their only infecting pathogen. The most common presenting coinfections were identified in 83 (28.1%) patients with *Mycoplasma pneumoniae*.

A total of 295 consecutive patients with a confirmatory diagnosis of COVID-19 were enrolled during the study period. Among them, 288 (97.6%) had a confirmatory diagnosis by PCR techniques validated by the Peruvian National Institute of Health. The seven patients left (2.4%), were diagnosed with a positive IgM result by ELISA in addition to suggestive symptoms. Figure 1 shows the coinfections reported in our study and we could observe that 141 (47.8%) patients had SARS-CoV-2 as their only infecting pathogen. 

The most common presenting coinfections were identified in 83 (28.1%) patients with *Mycoplasma pneumoniae*, 26 (8.8%) patients with *Chlamydia pneumoniae* and 34 (11.5%) patients with both bacteria. Adenovirus was identified in five (1.7%) patients, *Mycoplasma pneumoniae* + Adenovirus in two patients, *Chlamydia pneumoniae* + Adenovirus in two patients and RSV-B and *Chlamydia pneumoniae* in one patient. Finally, a combination *of Mycoplasma pneumoniae*, *Chlamydia pneumoniae* and Adenovirus was presented in one patient.

Table 1 shows the demographical and basal characteristics of the patients included, according to the pathogens identified. The mean age of the patients was 58 ± 14.0 years and 209 (70.9%) were male. Regarding past medical history, the two most common comorbidities found were hypertension (26.8%) and diabetes mellitus (22.3%). The most common clinical signs and symptoms on admission were cough (72.9%), dyspnea (69.8%) and fever (61.0%), which had a similar frequency in the different groups of coinfections. The group of patients who had the total number of coinfections had less expectoration compared to those with no coinfections (5.8% vs. 12.8%)

Table 2 shows the laboratory parameters and treatments that patients included received during hospitalization. However, no differences were observed in laboratory parameters among the different study groups. 

The clinical outcomes of the patients were evaluated in all study groups. The group of patients with total coinfections were more likely to develop sepsis than those patients without coinfection. Among the most relevant data, the group of coinfected had more superinfection events than those not coinfected (6.5 vs. 3.6%), a higher number of cases of heart failure (11.0 vs. 5.7%), as well as a mean number of days in ICU (16 vs. 8 days) and mechanical ventilation (16 vs. 9 days). In the coinfection between SARS-CoV-2 + *Mycoplasma pneumoniae*, which was the most frequently found, the highest number of cases of sepsis (37.4%) occurred. Frequency of ARDS in SARS-CoV-2 mono infection was 17.7%. Mortality was similar among all study groups, as shown in Table 3. 

**Figure 2:**
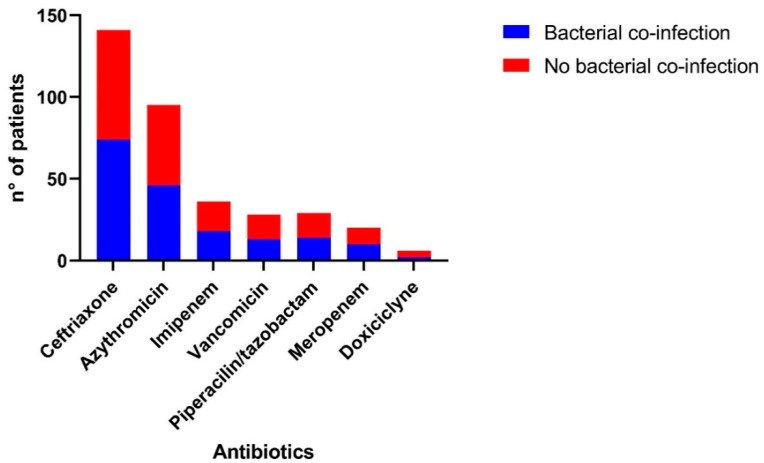
Administration of antibiotics in patients with and without bacterial coinfection. It was found that about half of the antibiotic prescriptions were given to patients who were not infected by any of the bacterial pathogens studied.

Finally, an evaluation of the antibiotics prescribed was carried out. We could highlight that the majority of patients were administered an antibiotic (69.5%). The most frequently antibiotics were ceftriaxone in 143 patients, azithromycin in 95 patients and imipenem in 36 patients. We could identify that nearly half of antibiotic prescriptions were given to patients that were not infected by any bacterial pathogen (Figure 2), while 41% (*n* = 39) of the patients who received macrolides empirically were PCR-positive for *Mycoplasma pneumoniae* and *Chlamydia pneumoniae*. 

## 3. Discussion

In this study, more than 50% of the patients evaluated with COVID-19 upon admission presented coinfection with other respiratory pathogens. These findings differ from those reported in the meta-analysis by Lansbury et al. [12] and Langford et al. [13], in which lower frequencies of coinfection were obtained in hospitalized patients (7% and 5.9%) and in critical patients (14% and 8.1%), respectively. The estimated proportion of coinfection in patients with COVID-19 varied according to the study site, season, clinical condition and diagnostic assays used [12,13,14,15].

Data on coinfections with SARS CoV-2 come mainly from studies carried out in China, United States and Spain [12,13,14]. We present the largest study in Peru including patients with moderate/severe COVID-19 pneumonia and coinfection with other respiratory pathogens. There are few reports in South America of cases of coinfection in patients upon admission. For example, Vial et al. [16] reported one case of coinfection with *Streptococcus pneumoniae* in Chile. In Brazil, it was documented that one patient presented *Lautropia*, *Prevotella,* and *Haemophilus* [17]. Finally, Orozco-Hernández et al. reported a case of coinfection with rhinovirus and enterovirus in Colombia [18].

The most common pathogen identified causing coinfections was *Mycoplasma pneumoniae*. In the current study, we used PCR to identify this microorganism, since it is highly sensitive and specific during the initial phase of infection [19,20,21], while serological techniques that detect IgM antibodies against *Mycoplasma pneumoniae* used in other reports in COVID-19 [12,21] may have less sensitivity in adult patients. In this age group, there is a weak antibody response and there is a need to take paired samples with documentation of elevated IgG titers to determine their clinical significance [20]. On the other hand, a study that evaluated IgM against *M. pneumoniae* determined 56.8% coinfection with SARS-CoV-2, a value above our findings [22].

In the current study, we found a total of 34 cases with simultaneous coinfection of *Mycoplasma pneumoniae* and *Chlamydia pneumoniae,* which was higher than in previous reports [23]. These bacteria have been reported to cause coinfections with other viruses; for example, it has been evidenced that a great frequency of bacterial coinfections are observed in patients with influenza [24] and it also is noteworthy that both *Mycoplasma pneumoniae* and *Chlamydia pneumoniae* were identified as coinfecting microorganisms in patients with SARS and MERS [25,26]. 

The impact of these findings in the adult population is not clear; however, coinfected patients presented a lower proportion of expectoration upon admission compared to non-coinfected patients. According to the score used by the Japanese Respiratory Society (JRS), cough without expectoration is one of the six criteria used to predict atypical pneumonia, with a sensitivity that reaches 83% [27,28]. We did not find differences in the leukocyte count between the groups of the total of coinfected and non-coinfected patients; nonetheless, it has been reported that leukopenia can be considered another diagnostic criterion to identify infections by atypical bacteria [28].

The majority of patients with COVID-19 presented fever, cough and dyspnea. These symptoms were similar among all study groups, which made the clinical differentiation difficult between COVID-19 monoinfections and coinfections with other pathogens [23]. In addition, the differentiation can be challenging in patients older than 60 years, in whom any respiratory infection may resemble typical bacteria pneumonia [29]. It has been proposed that pathogens such as *Mycoplasma pneumoniae* can exacerbate clinical symptoms, increase morbidity and prolong the stay in the ICU [30]. Our results also showed some differences between patients with monoinfection and coinfections, such as admission to the ICU, days in the ICU, mechanical ventilation. Mortality was similar among study groups. Another study found that patients with coinfection (COVID-19 and *Mycoplasma*) had higher mortality compared to patients with only COVID-19 disease [22].

The proportion of coinfections with other respiratory viruses was low, similar to other reports [12,31]. The most common viruses identified in our study were Adenovirus (HAdv) and only one case of respiratory syncytial virus B. According to an analysis carried out by the Pan American Health Organization, the distribution of other respiratory viruses did not exceed 5% in Peru during the pandemic [32]. Previous studies in Peru have reported a lower percentage of respiratory infections due to HAdv in people over 18 years of age, without specific characteristics that differentiate their presentation from other respiratory viruses [33].

We did not detect cases of coinfection with influenza viruses despite conducting the study during the winter months, during which this virus increases its incidence. This can be explained by social distancing and confinement orders that reduced the transmission of other respiratory viruses, including influenza and respiratory syncytial virus [34]. Another study in Peru reported cases of coinfection between SARS CoV-2 and influenza A (*n* = 4) and B (*n* = 1). While SARS-CoV-2 was identified by RT-PCR, influenza A and B were identified by indirect immunofluorescence (IFI) [35]. This fact represents a limitation of the study, given the lower diagnostic performance of IFI compared to PCR in the identification of respiratory pathogens [36]. 

In these coinfections, additional symptoms such as odynophagia and nasal congestion were described, with no additional complications [35]. A “synergistic effect” has been documented between influenza virus and COVID-19 that may increase the risk of mortality by almost two times, mainly in the elderly [37]. However, in the current study, we could not conclude that patients with another concurrent viral infection had a worse prognosis than patients with only SARS CoV-2 detection.

We considered that although it was not possible to document the proportion of patients who received the seasonal influenza vaccine, the proportion should be low, since in the place where the study was carried out, the vaccines were available at the end of April and the beginning of May and its application was not mandatory [38]. Social distancing measures, the massive use of masks, the closure of schools and other established biosafety measures may have reduced the transmission of other respiratory viruses.

We observed a higher proportion of sepsis in those patients with coinfections compared to monoinfection. SARS-CoV-2 has been reported to induce viral sepsis associated with secondary organ dysfunction in 25% and 83% of COVID-19 patients hospitalized in general services and critical care units, respectively [39,40]. Possible mechanisms proposed are increase in bacterial adherence, cellular destruction by viral enzymes, reduction of mucociliary clearance, reduction in chemotaxis, reduction in surfactant levels, dysbiosis of the microbiome, dysregulation of immune response and bacterial–viral synergism, among others [41].

We evaluated the use of antibiotics in our study and found that 205 (69.5%) patients received antibiotics upon admission. A previous meta-analysis found a similar proportion of antibiotic prescriptions among the studies (71.8%), in which the predominant antibiotics were quinolones and third-generation cephalosporins, comprising approximately 74% of the total antibiotics administered [13]. In our study, the most commonly antibiotics found were ceftriaxone in 143 patients, azithromycin in 95 patients and imipenem in 36 patients. This was because the so-called “respiratory” fluoroquinolones are restricted for the treatment of tuberculosis in Peru; therefore, third-generation cephalosporins and macrolides predominated in our study. We could observe that nearly 50% of the patients that received antibiotics did not have a bacterial coinfection and 41% of the patients who received azithromycin during hospitalization were coinfected with some atypical bacteria; however, the impact on the persistence of symptoms after antibiotic treatment could not be determined [42].

Routine administration of antibiotics is currently not indicated in the context of COVID-19 infection and may only be considered in the case of high clinical suspicion [43,44]. In addition, recently recommendations against empirical use of azithromycin in mild COVID-19 has been reported [45]. We are unaware of the future impact in our setting of the massive use of antibiotics after the pandemic. 

Although it is necessary to document the prevalence and possible resistance mechanisms of *Mycoplasma pneumoniae* and *Chlamydia pneumoniae* in Peru [46], the local rate of resistance to macrolides in *Streptococcus pneumoniae* strains obtained in hospitalized patients in Lima was higher than 30% [47]. Future studies are required to determine the role of antibiotics in inpatient COVID-19 care, as well as resistance rates, following the pandemic era. 

Our study had limitations. First, the percentage of coinfections at the beginning of hospitalization could be higher in relation to the number of respiratory pathogens evaluated if other multipathogenic molecular platforms were used (e.g., FilmArray); however, our institution does not have these tests for routine use. Despite this, the percentage of coinfections obtained exceeded that reported in the literature and the frequency of bacterial infections by atypical microorganisms that was obtained in an adult population occurred in established severity groups. Second, although a high frequency of coinfections was found, longitudinal studies must be carried out throughout the course of the disease to identify possible mixed infections through methods such as whole genome sequencing and to identify possible resistance mechanisms in pathogens such as *Mycoplasma pneumoniae* and *Chlamydia pneumoniae*. Third, the results obtained could not be extrapolated to other centers in Peru; however, it is possible that clusters of *M. pneumoniae* could circulate during the pandemic in Peru, for which it is necessary to use molecular and strain typing techniques to characterize these events. 

## 4. Materials and Methods

### 4.1. Study Design

A descriptive study was conducted on hospitalized patients with a confirmed diagnosis of moderate/severe pneumonia due to SARS-CoV-2 infection (molecular test or confirmation according to definitions established by the Peruvian Ministry of Health). The selection criteria included patients older than 18 years who were admitted to the Guillermo Almenara Irigoyen Hospital in Lima, Peru during the period of July–November 2020. The total number of hospitalized patients with a confirmed diagnosis of moderate/severe pneumonia due to SARS-CoV-2 infection in the hospital during the enrollment period was 660 patients. The selection was consecutive until 295 patients were enrolled and coincided with the highest peak of the first wave of the pandemic in Peru. The informed consent was signed upon admission to hospitalization. Only patients who accepted to be enrolled in this study and signed an informed consent were included. The patients not enrolled in the study were minors, pregnant women, patients who refused to participate and patients admitted to shifts when the personnel in charge of enrollment for the study were not present. 

### 4.2. Definitions

Moderate pneumonia was considered as follows: adult with clinical signs of pneumonia (fever, cough, dyspnea, respiratory distress) but no signs of severe pneumonia, including oxygen saturation ≥90% in room air. Severe pneumonia included patients with clinical signs of pneumonia (fever, cough, dyspnea, respiratory distress) plus one of the following: respiratory rate >30/min, severe respiratory distress, or oxygen saturation <90% in room air [48]. The radiological severity scored was assessed according to the study by Ho Yuen et al. [49].

### 4.3. Sampling and Nucleic Acids Extraction

Nasopharyngeal swab samples were collected from patients hospitalized in COVID-19 hospitalization wards and in the intensive care unit (ICU) within 48 h of hospital admission. RNA/DNA extraction was performed from 140 μL of the aliquoted samples. The QIAGEM^®^ QIAamp Genetic Material Isolation Kit was used according to the manufacturer’s instructions; 80 µL of RNA/DNA eluted was obtained after extraction and then continued with the amplification process.

Different viral and bacterial pathogens were evaluated by polymerase chain reaction (PCR), including: influenza A and B, respiratory syncytial virus (RSV), Adenovirus, *Mycoplasma pneumoniae,* and *Chlamydia pneumoniae*. Molecular diagnostic methods were carried out in the molecular biology laboratory of the Universidad Peruana de Ciencias Aplicadas (UPC).

### 4.4. Reverse Transcriptase Polymerase Chain Reaction (RT-PCR) for the Analysis of Respiratory Viruses

For the analysis of influenza A and influenza B, the primers and probes used were described by Carra et al. [50] and Selvaraju et al. [51], respectively. For the analysis of RSV-A and RSV-B, primers and probe were as described by Liu et al. [52] and for adenovirus, as described by Heim et al. [53]. For RNA viruses, a one-step RT-PCR was performed using TaqMan with a BHQ quencher probe at 125 nM and 250 nM of primers in a final volume of 20 mL. Then, 5 microliters of the extracted RNA was combined with 15 mL of the Ready RNA Virus Master (Roche Diagnostics, Mannheim, Germany). The amplification conditions for influenza A and influenza B were 50 °C for 10 min, followed by 45 cycles of 95 °C for 5 s, 57 °C for 15 s and 72 °C for 15 s; in the case of RSV-A and RSV-B, they were 50 °C for 10 min, followed by 50 cycles of 95 °C for 5 s, 51 °C for 20 s and 72 °C for 20 s. In the case of Adenovirus, the Fast Start DNA Master enzyme (Roche Diagnostics, Mannheim, Germany) was used and the amplification conditions were 50 °C for 10 min followed by 60 cycles of 95 °C for 5 s, 64 °C for 5 s and 72 °C for 15 s. All procedures were performed in a LightCycler 2.0 instrument and data were analyzed with LightCycler software 4.1 (Roche Diagnostics, Mannheim, Germany). 

### 4.5. Conventional Polymerase Chain Reaction (PCR) for Atypical Bacteria Mycoplasma pneumoniae and Chlamydia pneumoniae

For the amplification of atypical bacteria, primers and conditions previously described by Valle et al. [46] were used. The amplification consisted of an initial incubation at 95 °C for 2 min, followed by 40 cycles of 95 °C for 30 s; 58 °C for 30 s and 72 °C for 30 s; with a final extension at 72 °C for 5 min. Amplified sequences of 275 and 126 base pairs were detected for *Mycoplasma pneumoniae* and *Chlamydia pneumoniae,* respectively, visualized under agarose gel electrophoresis and nucleic acid staining (SybrGreen, Promega).

### 4.6. Data Analysis

For data and variables collection, the hospital electronic clinical charts were used. The data was obtained upon discharge of the patient and the information obtained was compiled in a database stored in the Excel v.2016 program. For the data analysis, no personal identifiers were considered. Descriptive statistics were performed and for the analysis of clinical results, the group of patients with COVID-19 monoinfection was compared versus the group that encompassed all evaluated coinfections. In addition, specific coinfections with the different pathogens were described separately. All calculations were performed using STATA Software version 15.0 for Windows (College Station, TX, USA). Graphics were created with GraphPad Prism 9.0.0.

## 5. Conclusions

Our study identified *Mycoplasma pneumoniae* and *Chlamydia pneumoniae* as the main microorganisms associated with coinfections in COVID-19 patients admitted to a referral hospital. Regarding respiratory viruses, Adenovirus and RSV-B were identified less frequently than atypical bacteria. Furthermore, the presence of multiple coinfections could be described in some patients. In the hospital setting, a higher proportion of sepsis, superinfections, stay in the ICU and mechanical ventilation was found in coinfected patients. Finally, a high proportion of patients received antibiotics, even in the absence of bacterial infections. Future studies are required to determine the role of other respiratory pathogens in COVID-19 and guide the rational use of antibiotics.

## Figures and Tables

**Table 1:** Demographic characteristics and symptoms on admission of patients with SARS CoV-2 and coinfections.

	Total (*n* = 295)	SARS-CoV-2 All Coinfections Evaluated (*n* = 154)	SARS-CoV-2 Monoinfection (*n* = 141)	SARS-CoV-2 + Adenovirus (*n* = 5)	SARS-CoV-2 + *Mycoplasma pneumoniae* (*n* = 83)	SARS-CoV-2 + *Chlamydia pneumoniae* (*n* = 26)	SARS-CoV-2 + *Mycoplasma pneumoniae*+ *Chlamydia pneumoniae* (*n* = 34)	Others (*n* = 6)
	Gender							
Male	209 (70.9%) [65.3–75.9%]	112 (72.2%) [64.9–79.5%]	97 (68.7%) [60.5–76.3%]	5 (100.0%)	60 (72.3%) [61.4–81.5%]	16 (61.5%) [40.6–79.7]	26 (76.5%) [58.8–89.2%]	5 (83.3%) [35.9–99.6%]
Female	86 (29.1%) [24.0–34.6%]	42 (27.8) [20.4–35.0%]	44 (31.2%) [23.7–39.5%]	0 (0.0%)	23 (27.7%) [18.4–38.6%]	10 (38.5%) [20.2–59.4%]	8 (23.5%) [10.7–41.1]	1 (16.6%) [0.4–64.1%]
	Age							
Mean/SD	58.0 ± 14.0	58.3 (13.8)	57.7 ± 14.3	59.6 ± 10.0	60.0 ± 13.7	55.8 ± 13.0	55.7 ± 15.6	59.67 ± 10.44
	Comorbidities							
Hypertension	79 (26.8%) [21.8–32.2%]	48 (31.1%) [23.9–39.1%]	31 (22.0%) [15.4–29.7%]	2 (40.0%) [5.3–85.3%]	26 (31.3) [21.6–42.4%]	7 (27.0%) [11.5–47.7%]	11 (32.3%) [17.4–50.5%]	2 (33.3%) [4.3–77.7]
Diabetes	66 (22.4%) [17.7–27.5%]	36 (23.4%) [16.9–30.8%]	30 (21.3%) [14.8–28.9%]	2 (40.0%) [5.3–85.3%]	22 (26.5%) [17.4–37.3%]	6 (23.1%) [8.9–43.6%]	6 (17.7%) [6.7–34.5%]	0 (0.0%)
Obesity	55 (18.6%) [14.4–23.6%]	24 (15.6%) [10.2–22.2%]	31 (22.0%) [15.4–29.7%]	0 (0.0%)	11 (13.3%) [6.8–22.5%]	5 (19.2%) [6.6–39.4%]	7 (20.6%) [87.0–37.9%]	1 (16.7%) [0.4–64.1%]
Asthma	12 (4.0%) [2.1–6.9%]	7 (4.5%) [1.8–9.1%]	5 (3.6%) [1.1–8.1%]	1 (20.0%) [0.5–71.6%]	2 (2.4%) [0.3–8.4%]	3 (11.5%) {24.4–30.2%]	1 (2.9%) [0.1–15.3%]	0 (0.0%)
Coronary artery disease	12 (4.1%) [2.1–6.9%]	4 (2.6%) [0.7–6.5%]	8 (5.7%) {2.4–10.8%]	0 (0.0%)	3 (3.6%) [0.7–10.2%]	1 (3.9%) [0.1–19.6%]	0 (0.0%)	0 (0.0%)
Cancer	7 (2.4%) [0.9–4.8%]	4 (2.6%) [0.7–6.5%]	3 (2.1%) [0.4–6.1%]	0 (0.0%)	4 (4.8%) [1.3–11.8]	0 (0.0%)	0 (0.0%)	0 (0.0%)
CKD*	4 (1.4%) [0.4–3.4%]	4 (2.6%) [0.7–6.5%]	0 (0.0%)	1 (20.0%) [0.5–71.6%]	0 (0.0%)	0 (0.0%)	2 (5.9%) [0.7–19.7%]	0 (0.0%)
Others	56 (19.0%) [14.7–23.9%]	28 (18.1%) [12.4–25.2%]	28 (19.9%) [13.6–27.4%]	0 (0.0%)	16 (19.3%) [11.4–29.4%]	4 (15.4%) [4.4–34.8%]	7 (20.6%) [8.7–37.9%]	1 (16.6%) [0.4–64.1%]
	Symptoms							
Cough	215 (72.9%) [67.4–77.8%]	107 (69.5%) [61.5–76.6%]	108 (76.6%) [68.7–83.3%]	4 (80.0%) [28.3–99.4%]	57 (68.7%) [57.5–78.4%]	17 (65.3%) [44.3–82.7%]	24 (70.6%) [52.5–84.9%]	5 (833%) [35.9–99.6%]
Dyspnea	206 (69.8%) [64.2–75.0%]	105 (68.2%) [60.2–75.4%]	101 (71.6%) [63.4–78.9%]	3 (60.0%) [14.7–94.7%]	61 (73.5%) [62.7–82.6%]	15 (57.7%) [37.9–76.6%]	22 (64.7%) [46.5–80.2%]	4 (66.7%) [22.3–95.7%]
Fever	180 (61.0%) [55.2–66.6%]	95 (61.7%) [53.5–69.3%]	85 (60.3%) [51.7–68.4%]	4 (80.0%) [28.3–99.4%]	48 (57.8%) [46.5–68.5%]	17 (65.4%) [44.3–82.7%]	23 (67.7%) [49.5–82.6%]	3 (50.0%) [11.8–88.1%]
Fatigue	148 (50.2%) [44.3–56.0%]	74 (48.1%) [39.9–56.2%]	74 (52.5%) [43.9–60.9%]	4 (80.0%) [28.3–99.4%]	39 (47.0%) [35.9–58.2%]	13 (50.0%) [29.9–70.0%]	14 (41.2%) [24.6–59.3%]	4 (66.7%) [22.3–95.7%]
Odynophagia	39 (13.2%) [9.6–17.6%]	19 (12.3%) [7.6–18.5%]	20 (14.2%) [8.8–21.1%]	0 (0.0%)	11 (13.3%) [6.8–22.4%]	3 (11.5%) [2.4–30.1%]	4 (11.8%) [3.3–27.4%]	1 (16.7%) [0.4–64.1%]
Headache	35 (11.9%) [8.4–16.1%]	17 (11.0%) [6.5–17.0%]	18 (12.8%) [7.7–19.4%]	1 (20.0%) [0.5–71.6%]	5 (6.0%) [1.9–13.5%]	4 (15.4%) [4.4–34.8%]	6 (17.7%) [6.7–34.5%]	1 (16.7%) [0.4–64.1%]
Nausea/vomiting	18 (6.1%) [3.6–9.5%]	12 (7.8%) [4.1–13.2%]	6 (4.3%) [1.5–9.0%]	0 (0.0%)	8 (9.6%) [4.3–18.1%]	1 (3.9%) [0.1–19.6%]	2 (5.9%) [0.7–19.6%]	1 (16.7%) [0.4–64.1%]
Diarrhea	20 (6.8%) [04.2–10.2%]	11 (7.1%) [3.6–12.4%]	9 (6.4%) [2.9–11.7%]	0 (0.0%)	6 (7.2%) [2.7–15.0%]	2 (7.7%) [0.9–25.1%]	2 (5.9%) [0.7–19.6%]	1 (16.7%) [0.4–64.1%]
Expectoration	27 (9.1%) [6.1–13.0%]	9 (5.8%) [2.7–10.8%]	18 (12.8%) [7.7–19.4%]	0 (0.0%)	6 (7.2%) [2.7–15.0%]	1 (3.9%) [0.1–19.6%]	1 (2.9%) [0.1–15.3%]	1 (16.7%) [0.4–64.1%]
Anosmia	11 (3.7%) [1.8–6.6%]	5 (3.3%) [1.1–7.4%]	6 (4.3%) [1.5–9.0%]	0 (0.0%)	3 (3.6%) [0.7–10.2%]	1 (3.9%) [0.1–19.6%]	1 (2.9%) [0.1–15.3%]	0 (0.0%)
Days since symptom onset *	7 (5–10)	7 (5–10)	7 (6–10)	6 (3–9)	7 (4–10)	7 (6–9)	7 (5–13)	7 (6–12)
CURB 65 *	1 (0–2)	1 (0–2)	1 (0–2)	1 (0–3)	1 (0–2)	0 (0–1)	1 (0–1)	1 (0–2)

Others included: *Mycoplasma pneumoniae* + Adenovirus (*n* = 2), *Chlamydia pneumoniae* + Adenovirus (*n* = 2), VRS-B + *Chlamydia pneumoniae* (*n* = 1) and *Mycoplasma pneumoniae* + *Chlamydia* *pneumoniae* + Adenovirus (*n* = 1). * Median (interquartile range); CKD = chronic kidney disease; * CURB 65: Severity Score for Community-Acquired Pneumonia; * SD = standard deviation. For each qualitative variable, the percentage and its respective 95% confidence interval are report.

**Table 2:** Laboratory parameters and radiological and treatment characteristics of patients with SARS-CoV-2 and coinfections.

	Total (*n* = 295)	SARS-CoV-2 All Coinfections Evaluated (*n* = 154)	SARS-CoV-2 Monoinfection (*n* = 141)	SARS-CoV-2 + Adenovirus (*n* = 5)	SARS-CoV-2 + *Mycoplasma pneumoniae* (*n* = 83)	SARS-CoV-2 + *Chlamydia pneumoniae* (*n* = 26)	SARS-CoV-2 + *Mycoplasma pneumoniae+ Chlamydia pneumoniae* (*n* = 34)	Others (*n* = 6)
	Laboratory parameters *							
Hemoglobin (g/dl)	14.20 (13.1–15.4)	14.5 (13.2–15.4)	14 (12.9–15.5)	14 (12.2–16.3)	14.5 (13.1–15.4)	14.1 (13.1–14.8)	14.45 (13.3–15.6)	14.65 (13.2–16.2)
Leucocytes (× 10^9^ mL)	9.1 (7.9–12.3)	8.85 (7–11.9)	9.2 (7–12.3)	10.4 (5.25–14.05)	8.3 (6.4–11.4)	8.65 (7.3–11.5)	10.1 (7.3–12.8)	9.4 (8.1–13)
Lymphocytes (Absolute count)	820 (504–1290)	797 (518–1242)	847 (497.5–1325.5)	828 (445.5–1866)	888 (615–1348)	837 (495–1442)	653.5 (468–1020)	736 (486–872)
Platelets (× 10^9^ mL)	270 (202–350)	270.5 (204–342.5)	265 (192.5–355.5)	213 (148.5–312.5)	267 (201–340)	295 (218–333)	289 (225–394)	215 (197–232)
ALT (U/L)	49 (26.5–88)	50 (26–88)	45 (27–87)	45 (15.5–193)	50 (25–93)	47 (31.5–63)	56 (26–103)	60 (52–70)
Creatinine (mg/dL)	0.7 (0.6–0.9)	0.7 (0.6–0.9)	0.7 (0.6–0.9)	1 (0.7–1.2)	0.7 (0.6–0.8)	0.65 (0.5–0.9)	0.75 (0.6–0.9)	0.75 (0.5–1)
C-reactive protein (mg/L)	90 (56–210)	90 (58–191)	90 (54.2–235.1)	277.4 (NA)	89 (58.8–174)	72.7 (43–226)	90 (62.7–201.75)	181 (NA)
LDH (U/L)	298 (242.5–378.5)	307 (251–376)	281.5 (233–381)	428 (NA)	299 (243.5–364)	331.5 (24.5–366)	291 (244–387)	368 (333.5–433)
Procalcitonin (ng/mL)	0.09 (0.06–0.25)	0.14 (0.07–0.27)	0.09 (0.04–0.18)	0.16 (NA)	0.09 (0.075–0.64)	0.13 (0.065–0.22)	0.23 (0.11–1.16)	0.1 (NA)
D-Dimer (µg/mL)	0.66 (0.39–1.2)	0.7 (0.3–1.2)	0.6 (0.39–1.22)	0.87 (NA)	0.8 (0.45–0.98)	0.675 (0.24–1.105)	0.465 (0.35–1.915)	0.725 (0.36–1.39)
Troponin (ng/mL)	0.006 (0.001–0.10)	0.006 (0.001–0.01)	0.006 (0.003–0.01)	0.011 (NA)	0.006 (0.001–0.01)	0.019 (0.008–0.149)	0.004 (0.001–0.1)	0.006 (NA)
Ferritin (ng/mL)	664.5 (346–1220)	639 (346–1127)	712 (344–1238.5)	1260 (NA)	620.5 (330–1066.5)	455 (184–821)	748.5 (510–1387)	817.5 (239–1759)
CPK (U/L)	55 (33–88)	42 (31–78)	49 (34.5–90)	40 (NA)	40.5 (34–165)	42 (18–70)	45 (NA)	49 (NA)
PT (s)	10.9 (10.4–11.5)	10.8 (10.4–11.3)	11 (10.4–11.6)	10.8 (10.3–12.2)	10.9 (10.4–11.25)	10.7 (10.4–11.9)	10.9 (10.6–11.2)	10.25 (10.1–10.8)
	Radiological score							
Mean /SD	5.92 ± 1.55	5.90 ± 1.15	5.92 ± 1.86	6 ± 2	5.82 ± 1.90	5.46 ± 2.00	6.53 ± 1.58	5.67 ± 2.58
	Treatments							
Hydroxychloroquine	3 (1.0%) [0.2–29.4%]	1 (0.7%) [0.1–3.5%]	2 (1.4%) [0.2–5.0%]	0 (0.0%)	1 (1.2%) [0.1–6.5%]	0 (0.0%)	0 (0.0%)	0 (0.0%)
Ivermectin	24 (8.1%) [5.2–11.9%]	9 (5.8%) [2.7–10.8%]	15 (10.6%) [6.1–16.9%]	0 (0.0%)	7 (8.4%) [3.5–16.6%]	0 (0.0%)	1 (2.9%) [0.1–15.3%]	1 (16.7%) [0.4–64.1%]
Antibiotics	205 (69.5%) [63.8–74.6%]	110 (71.4%) [63.6–78.4%]	95 (67.4%) [58.9–75.0%]	4 (80.0%) [28.3–99.4%]	59 (71.1%) [60.1–80.5%]	18 (69.2%) [48.2–85.6%]	26 (76.5%) [58.8–89.2%]	3 (50.0%) [11.8–88.1%]
Dexamethasone	250 (84.7%) [80.1–88.7%]	137 (89.0%) [82.9–93.4%]	113 (80.1%) [72.6–86.4%]	5 (100.0%)	71 (85.5%) [76.1–92.3%]	22 (84.6%) [65.1–95.6%]	33 (97.1%) [84.7–99.9%]	6 (100.0%)
Methyilprednisolone	1 (0.3%) [0.1–18.7%]	0 (0.0%)	1 (0.7%) [0.1–3.9%]	0 (0.0%)	0 (0.0%)	0 (0.0%)	0 (0.0%)	0 (0.0%)
Hydrocortisone	2 (0.7%) [0.1–24.3%]	1 (0.7%) [0.1–3.5%]	1 (0.7%) [0.1–3.9%]	0 (0.0%)	0 (0.0%)	0 (0.0%)	1 (2.9%) [0.1–15.3%]	0 (0.0%)
Binasal cannula	161 (54.6%) [48.7–60.4%]	81 (52.6%) [44.4–60.6%]	80 (56.7%) [48.1–65.0%]	2 (40.0%) [5.3–85.3%]	44 (53.1%) [41.7–64.0%]	12 (46.2%) [26.6–66.6%]	19 (55.9%) [37.9–72.8%]	4 (66.7%) [22.3–95.7%]
Reservoir bag	111 (37.6%) [32.1–43.4%]	63 (40.9%) [33.1–49.1%]	48 (34.0%) [26.3–42.5%]	1 (20.0%) [0.5–71.6%]	33 (39.8%) [0.8–10.2%]	12 (46.2%) [26.6–66.6%]	15 (44.1%) [27.2–62.1%]	2 (33.3%) [4.3–77.7%]
High-flow nasal cannula	20 (6.8%) [4.2–10.3%]	12 (7.8%) [4.1–13.2%]	8 (5.7%) [2.5–10.9%]	2 (40.0%) [5.3–85.3%]	6 (7.2%) [2.7–15.0%]	1 (3.9%) [0.1–19.6%]	2 (5.9%) [0.7–19.6%]	1 (16.7%) [0.4–64.1%]
Mechanical ventilation	20 (6.8%) [4.2–10.3%]	10 (6.5%) [3.2–11.6%]	10 (7.1%) [3.5–12.6%]	0 (0.0%)	5 (6.0%) [1.9–13.5%]	3 (11.5%) [2.4–30.2%]	1 (2.9%) [0.1–15.3%]	1 (16.7%) [0.4–64.1%]
Norepinephrine	21 (7.1%) [4.5–10.7%]	8 (5.2%) [2.3–99.8%]	13 (9.3%) [5.0–15.52%]	1 (20.0%) [0.5–71.6%]	4 (4.8%) [1.3–11.8%]	2 (7.7%) [0.9–25.1%]	1 (2.9%) [0.1–15.3%]	0 (0.0%)
Epinephrine	3 (1.0%) [2.1–29.4%]	2 (1.3%) [0.2–4.6%]	1 (0.7%) [0.1–3.9%]	1 (20.0%) [0.5–71.6%]	1 (1.2%) [0.1–6.5%]	0 (0.0%)	0 (0.0%)	0 (0.0%)
Hemodialysis	3 (1.0%) [2.1–29.4%]	1 (0.7%) [0.1–3.5%]	2 (1.4%) [0.2–5.0%]	0 (0.0%)	0 (0.0%)	1 (3.0%) [0.1–19.6%]	0 (0.0%)	0 (0.0%)

Others included: *Mycoplasma pneumoniae* + Adenovirus (*n* = 2), *Chlamydia pneumoniae* + Adenovirus (*n* = 2), VRS-B + *Chlamydia pneumoniae* (*n* = 1) and *Mycoplasma pneumoniae* + *Chlamydia pneumoniae* + Adenovirus (*n* = 1). NA = not available; * Median (interquartile range); ALT = alanine transaminase; LDH = lactate dehydrogenase; CPK = creatine phosphokinase; PT = prothrombin time; * SD = standard deviation.

**Table 3:** Clinical outcomes in patients with COVID-19 and coinfections.

Clinical Outcomes	Total (*n* = 295)	SARS-CoV-2 All Coinfections Evaluated (*n* = 154)	SARS-CoV-2 Monoinfection (*n* = 141)	SARS-CoV-2 + Adenovirus (*n* = 5)	SARS-CoV-2 + *Mycoplasma pneumoniae* (*n* = 83)	SARS-CoV-2 + *Chlamydia pneumoniae* (*n* = 26)	SARS-CoV-2 + *Mycoplasma pneumoniae* + *Chlamydia pneumionie* (*n* = 34)	Others (*n* = 6)
Sepsis	80 (27.1%) [22.1–32.6%]	51 (33.1%) [25.7–41.1%]	29 (20.6%) [14.2–28.2%]	2 (40.0%) [5.3–85.3%]	31 (37.4%) [26.9–48.6%]	6 (23.1%) [8.9-43.6%]	9 (26.5%) [12.8–44.3%]	3 (50.0%) [11.8–88.1%]
ARDS	60 (20.3%) [15.9–25.4%]	35 (22.7%) [16.4–30.2%]	25 (17.7%) [11.8–25.1%]	2 (40.0%) [5.3–85.3%]	13 (15.7%) [8.6–25.3%]	9 (34.6%) [17.2–55.6%]	8 (23.5%) [10.7–41.1%]	3 (50.0%) [11.8–88.1%]
Heart failure	25 (8.5%) [5.6–12.3%]	17 (11.0%) [6.6–17.1%]	8 (5.7%) [2.4%-10.9%]	0 (0.0%)	7 (8.4%) [3.5–16.6%]	6 (23.1%) [8.9–43.6%]	3 (8.8%) [1.9–23.6%]	1 (16.7%) [0.4–64.1%]
Septic shock	24 (8.1%) [5.3–11.8%]	11 (7.1%) [3.6–12.4%]	13 (9.2%) [5.0–15.3%]	2 (40.0%) [5.3–85.3%]	5 (6.0%) [1.9–13.5%]	3 (11.5%) [2.4–30.2%]	1 (2.9%) [0.1–15.3%]	0 (0.0%)
Coagulopathy	17 (5,8%) [3.4–9.1%]	10 (6.5%) [3.2–11.6%]	7 (5.0%) [2.0–9.9%]	1 (20.0%) [0.5–71.6%]	4 (4.8%) [1.3–11.8%]	4 (15.4%) [4.4–34.8%]	1 (2.9%) [0.1–15.3%]	0 (0.0%)
Acute myocardial injury	12 (4.1%) [2.1–6.9%]	4 (2.6%) [0.7–6.5%]	8 (5.7%) [2.5–10.8%]	0 (0.0%)	3 (3.6%) [0.8–10.2%]	1 (3.9%) [0.1–19.6%]	0 (0.0%)	0 (0.0%)
Superinfection	15 (5.1%) [2.8–8.5]	10 (6.5%) [3.2–11.6%]	5 (3.6%) [1.2–8.1%]	1 (20.0%) [0.5–71.6%]	5 (6.0%) [1.9–13.5%]	3 (11.5%) [2.4–30.2%]	1 (2.9%) [0.01–15.3%]	1 (16.7%) [0.4–64.1%]
Acute kidney injury	30 (10.2%) [6.9–14.1%]	16 (10.4%) [6.1–16.3%]	14 (9.9%) [5.5–16.1%]	1 (20.0%) [0.5–71.6%]	6 (7.2%) [2.7–15.1%]	4 (15.4%) [4.4–34.8%]	4 (11.8%) [3.3–27.4%]	1 (16.67) (0.4–64.1%)
Respiratory acidosis	28 (9.5%) [6.4–13.4%]	13 (8.4%) [4.6–14.0%]	15 (10.6%) [6.1–16.9%]	1 (20.0%) [0.5–71.6%]	8 (9.6%) [4.3–18.1%]	2 (7.7%) [0.9–25.1%]	1 (2.9%) [0.1–15.3%]	1 (16.7%) [0.4–64.1%]
ICU Admission	29 (9.8%) [6.6–13.8%]	17 (11.4%) [6.6–17.1%]	12 (8.5%) [4.5–14.4%]	1 (20.0%) [0.5–71.6%]	10 (12.5%) [5.9–21.0%]	3 (11.5%) [2.4–30.2%]	2 (5.9%) [0.7–19.7]	1 (16.7%) [0.4–64.1%]
Days in ICU	11 (6–21)	16 (6–19)	8 (5.5–21)	17 (NA)	13.5 (6–25)	18 (NA)	35.5 (NA)	1 (NA)
Days in mechanical ventilation	11 (1–19.5)	16 (1–19)	9 (7–20)	17 (NA)	11 (1–21)	18 (NA)	25 (NA)	1 (NA)
Hospitalization days	10 (7–15)	10 (7–15)	10 (7–15)	7 (5.5–17.5)	11 (7–15)	10.5 (6–21)	9.5 (7–15)	8 (7–15)
Death	59 (20.0%) [15.5–25.0%]	32 (20.8%) [14.7–28.0%]	27 (19.2%) [13.0–26.6%]	2 (40.0%) [5.3–85.3%]	15 (18.1%) [10.5–28.0%]	6 (23.1%) [8.9–43.6%]	6 (17.7%) [6.7–34.5%]	3 (50.0%) [11.8–88.1%]

Others included: *Mycoplasma pneumoniae* + Adenovirus (*n* = 2), *Chlamydia pneumoniae* + Adenovirus (*n* = 2), VRS-B + *Chlamydia pneumoniae* (*n* = 1) and *Mycoplasma pneumoniae* + *Chlamydia pneumoniae* + Adenovirus (*n* = 1). ARDS = acute respiratory distress syndrome; ICU = intensive care unit. For each qualitative variable, the percentage and its respective 95% confidence interval are reported.

## Data Availability

The data supporting the reported results are available from the corresponding author upon reasonable request.
